# Supplementary material for: Feasible future global scenarios for human life evaluations
Source: Nat Commun. 2019 Jan 11;10:161. doi: 10.1038/s41467-018-08002-2 (PMC6329754; doi:10.1038/s41467-018-08002-2)
Supplement: Supplementary file 1 — Supplementary Information [file 41467_2018_8002_MOESM1_ESM.pdf]

Supplementary Information

# Feasible future global scenarios for human life evaluations

Christopher Barrington-Leigh, Eric Galbraith

Published in *Nature Communications, 2019*

## Supplementary Figures

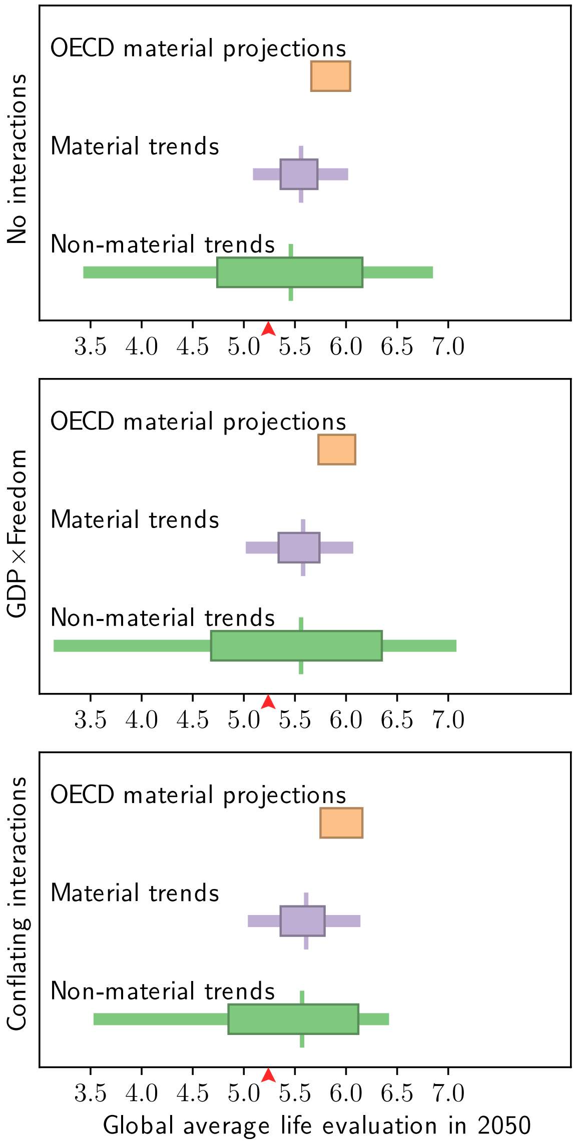


Supplementary Figure 1: **Projections using alternate model specifications incorporating interaction effects.** The top panel is based on our preferred model, while the second and third show projections made using alternative specifications (4) and (8) in Supplementary Table 8.

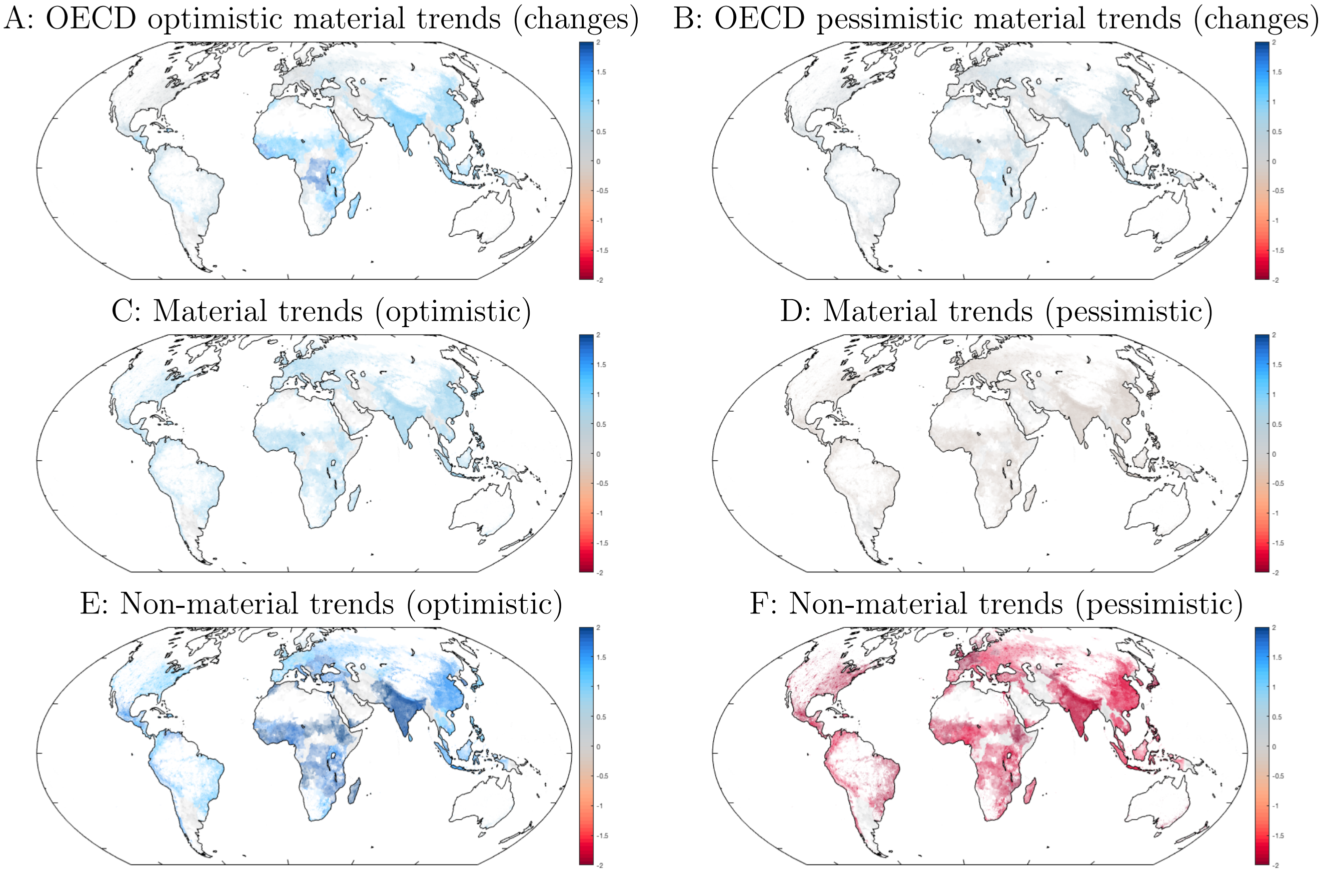


Supplementary Figure 2: **Geographic distribution of changes in life evaluation (2050 scenarios, 2P coefficients)**. Projections based on two-period (2P) coefficients.

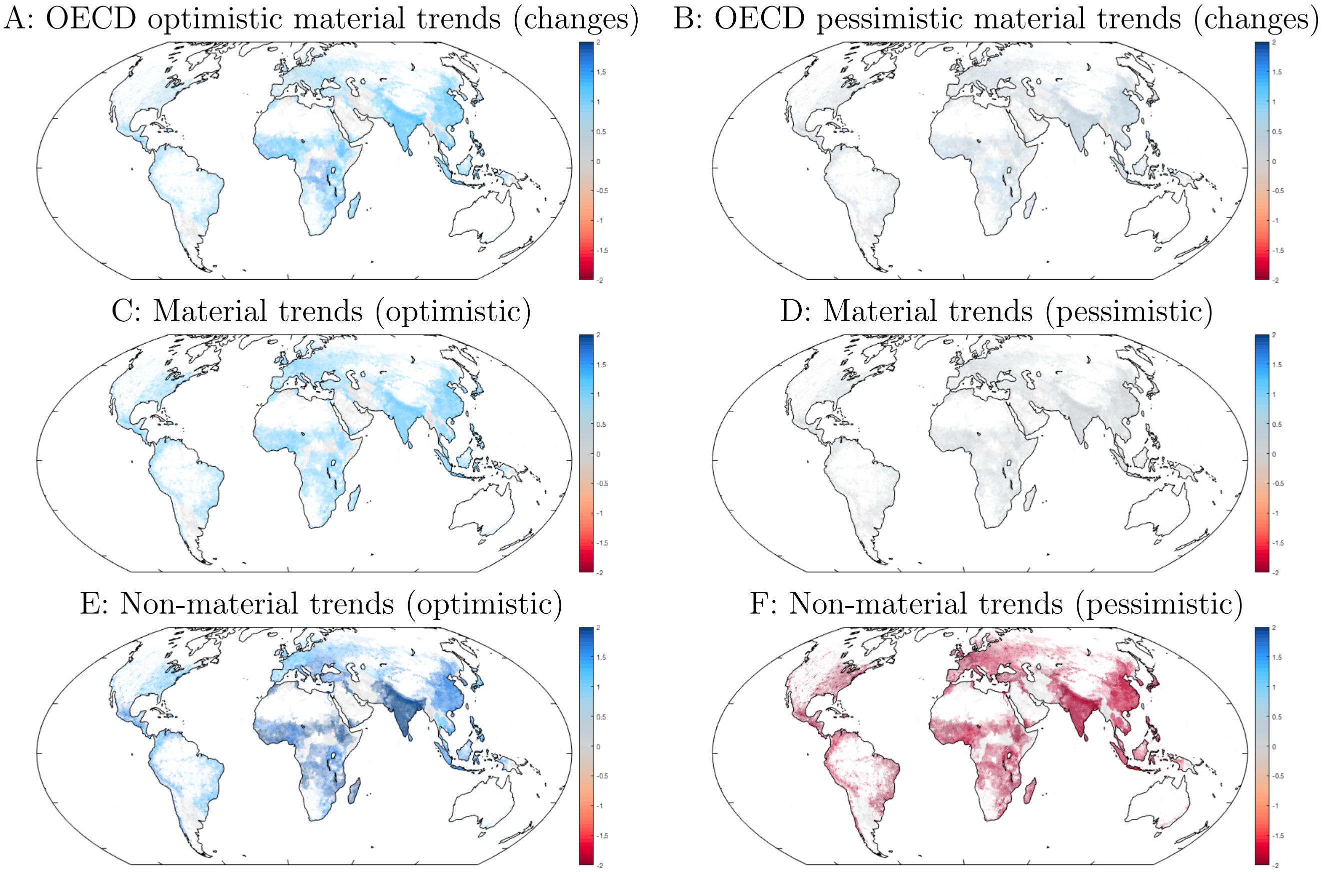


Supplementary Figure 3: **Geographic distribution of changes in life evaluation (2050 scenarios, XS coefficients)**. Projections based on cross-sectional (XS) coefficients.

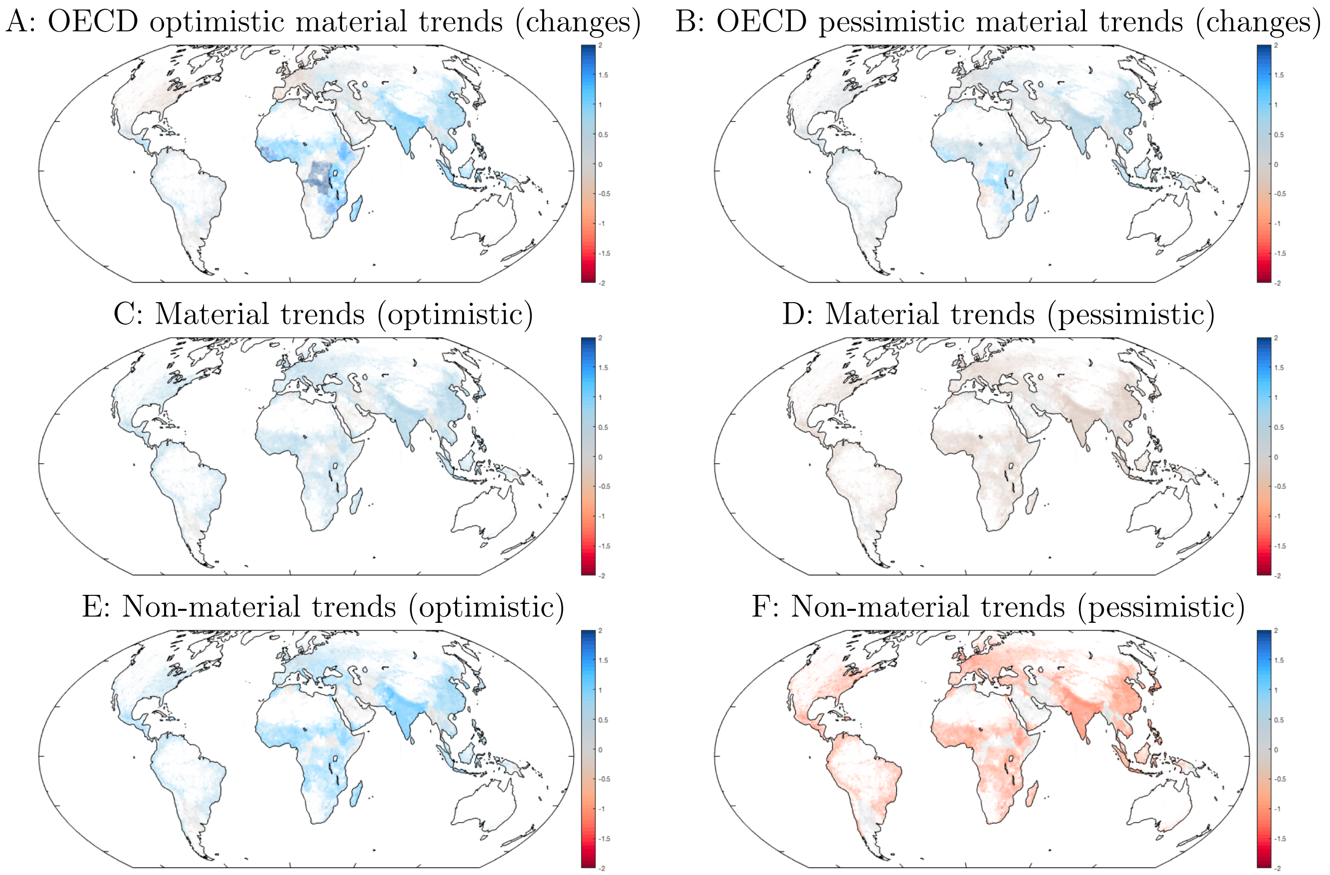


Supplementary Figure 4: **Geographic distribution of changes in life evaluation (2050 scenarios, FE coefficients)**. Projections based fixed-effect (FE) coefficients.

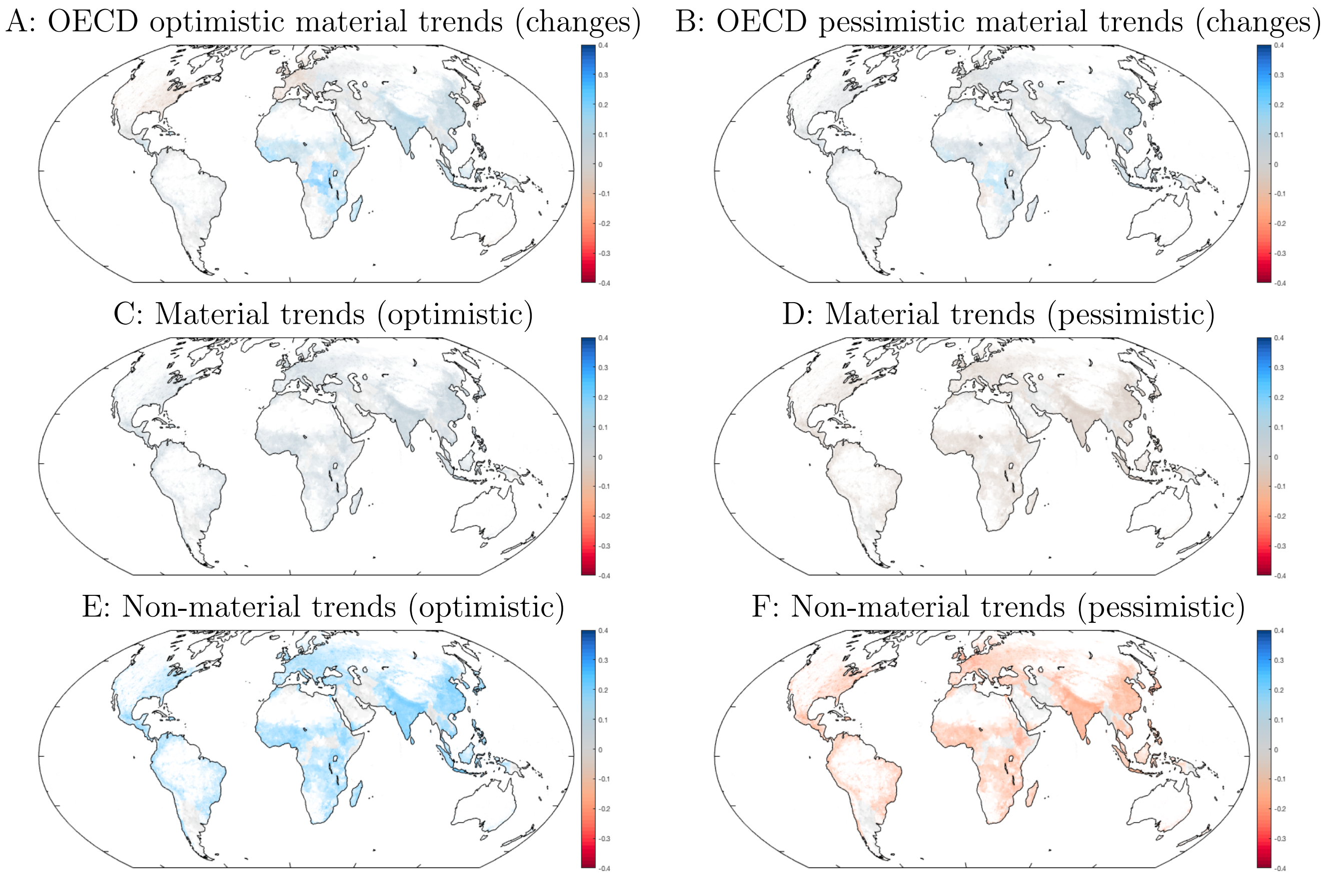


Supplementary Figure 5: **Geographic distribution of changes in affect balance (2050 scenarios, 2P coefficients)**. Projections based on two-period (2P) coefficients.

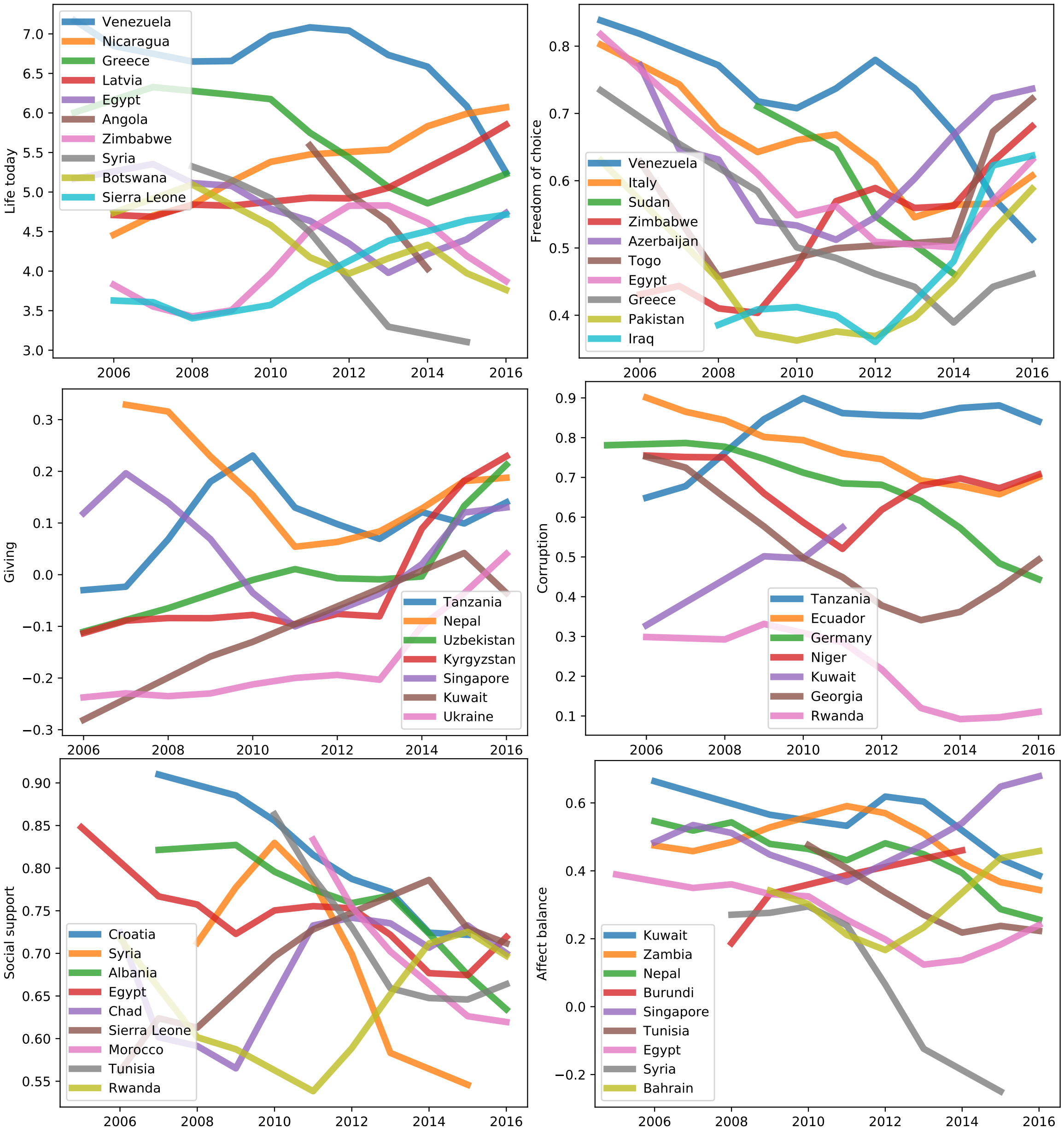


Supplementary Figure 6: **Strong trends in life evaluation, affect balance, and predictor variables.** Values are smoothed with a 3-year rolling average. Countries listed are those with the largest changes, in each case, during the observation period. These developments are shown for illustration only; the trends used for our projections are taken from the full distribution across countries of average rates of change over the entire period.

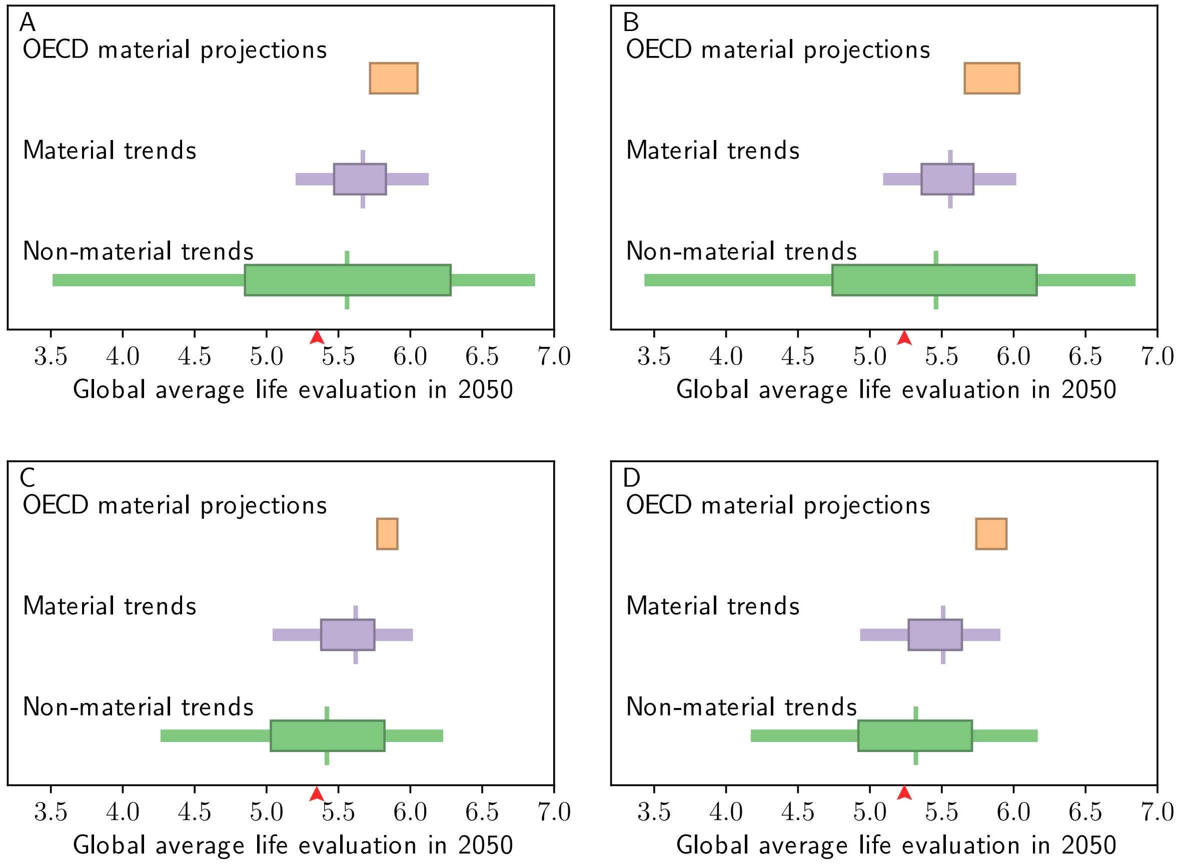


Supplementary Figure 7: **Range of scenario outcomes for global mean life evaluations in 2050**. (A) and (C) show mean life evaluation across countries, on a 0–10 scale , while (B) and (D) show population-weighted global means. (A) and (B) are calculated using our estimated coefficients in the two-period model, while (C) and (D) are from our fixed effects model. In each panel, the coloured bars show the ranges of feasible life evaluations attributable solely to changes spanning the pessimistic to optimistic scenarios for the each of the nonmaterial trends (green), material trends (orange), and OECD factors (blue).

## Supplementary Tables

| | Country-weighted | | Pop'n-weighted | | | | | |
|---|---|---|---|---|---|---|---|---|
| | $\mu$ | std | $\mu$ | std | min | max | 10%ile $\frac{d}{dy}$ | 90%ile $\frac{d}{dy}$ |
| $\delta$Life today | .027±.049 | .53 | −.21±.19 | .56 | −1.60 | 1.37 | −.099 | .086 |
| $\delta-$ affect | .020±.005 | .057 | .034±.017 | .056 | −.10 | .18 | −.005 | .018 |
| $\delta+$ affect | .002±.005 | .053 | .0004±.009 | .041 | −.14 | .13 | −.009 | .012 |
| $\delta$log(GDP/capita) | .16±.016 | .17 | .25±.063 | .18 | −.35 | .68 | −.004 | .047 |
| $\delta$Life expectancy | 2.6±.18 | 1.96 | 2.4±.19 | 1.43 | .53 | 13.4 | .12 | .51 |
| $\delta$Giving | −.003±.010 | .11 | .007±.017 | .092 | −.24 | .32 | −.018 | .017 |
| $\delta$Freedom of choice | .034±.009 | .095 | .038±.013 | .081 | −.30 | .24 | −.010 | .021 |
| $\delta$Corruption | −.021±.008 | .086 | −.030±.015 | .076 | −.34 | .17 | −.018 | .011 |
| $\delta$Social support | −.017±.006 | .061 | −.006±.010 | .048 | −.24 | .16 | −.012 | .010 |

Supplementary Table 1: **Summary statistics: two-period changes**. As in Supplementary Table 2, but showing changes between the mean level from 2005–2007 in each country, and its mean between 2014–2016. Country-weighted values show a 17% increase in GDP/capita (i.e., log increase of 0.16), a 2.6-year increase in life expectancy, a 1.7% decrease in affirmative responses to the social support question, and no significant change in life evaluations.

| | Country-weighted | | Pop'n-weighted | | | |
|---|---|---|---|---|---|---|
| | $\mu$ | std | $\mu$ | std | min | max |
| ΔLife today | .002±.015 | .39 | −.014±.031 | .34 | −2.00 | 1.65 |
| Δ− affect | .003±.002 | .047 | .004±.003 | .039 | −.17 | .27 |
| Δ+ affect | .001±.002 | .051 | .0002±.004 | .046 | −.22 | .20 |
| Δlog(GDP/capita) | .019±.001 | .039 | .036±.004 | .037 | −.44 | .21 |
| ΔLife expectancy | .29±.010 | .27 | .26±.010 | .19 | −.75 | 1.73 |
| ΔGiving | −.002±.002 | .070 | .0004±.007 | .071 | −.30 | .44 |
| ΔFreedom of choice | .005±.003 | .071 | .004±.009 | .076 | −.30 | .32 |
| ΔCorruption | −.004±.003 | .059 | −.003±.003 | .044 | −.27 | .25 |
| ΔSocial support | −.0007±.002 | .054 | .001±.005 | .050 | −.36 | .21 |

Supplementary Table 2: **Summary statistics: annual changes**. As in Supplementary Table 3, but showing statistics describing the annual changes across countries, generated by taking first differences in every case when a variable is observed in a country over two successive years. All values are given as the change per year.

| | Country-weighted | | Pop'n-weighted | | | | |
|---|---|---|---|---|---|---|---|
| | $\mu$ | std | $\mu$ | std | min | max | $N$ |
| Life today | 5.4±.037 | 1.15 | 5.3±.069 | .95 | 2.7 | 8.0 | 1190 |
| − affect | .26±.003 | .078 | .25±.007 | .071 | .10 | .70 | 1190 |
| + affect | .71±.003 | .11 | .73±.007 | .091 | .36 | .94 | 1190 |
| log(GDP/capita) | 9.1±.044 | 1.23 | 9.1±.058 | .96 | 6.4 | 11.7 | 1190 |
| Life expectancy | 61.0±.31 | 8.7 | 61.8±.51 | 7.2 | 36.2 | 76.4 | 1190 |
| Giving | −.0003±.005 | .16 | −.004±.013 | .16 | −.32 | .54 | 1190 |
| Freedom of choice | .72±.005 | .14 | .74±.008 | .12 | .26 | .98 | 1190 |
| Corruption | .75±.007 | .19 | .82±.009 | .12 | .035 | .98 | 1190 |
| Social support | .81±.004 | .12 | .76±.013 | .13 | .29 | .99 | 1190 |

Supplementary Table 3: **Summary statistics: annual time series**. For each subjective well-being metric and predictor variable, the mean ($\mu$) and standard deviation (std) among all countries are given (Country-weighted). The mean and standard deviation are also given after weighting the value in each country by its population, in order to estimate the average value for all humans (Pop'n-weighted). Also shown are the minimum (min) and maximum (max) among all countries in the dataset, as well as the total number of country-year observations (N).

| | Life today | | | | | | | | |
|---|---|---|---|---|---|---|---|---|---|
| | (1) | (2) | (3) | (4) | (5) | (6) | (7) | (8) | (9) |
| log(GDP/capita) | **.35**$^\dagger$ | **.93**$^\dagger$ | **1.17**$^\dagger$ | *.66*$^+$ | **.36**$^\dagger$ | **.96**$^\dagger$ | *.19*$^+$ | **.14**$^\dagger$ | **.095**$^\dagger$ |
| | (.062) | (.23) | (.30) | (.37) | (.065) | (.24) | (.11) | (.034) | (.024) |
| Social support | **2.2**$^\dagger$ | **1.38**$^\dagger$ | **1.05**$^\dagger$ | **2.6**$^\dagger$ | **.23**$^\dagger$ | **.15**$^\dagger$ | **.29**$^\dagger$ | **.14**$^\dagger$ | **.19**$^\dagger$ |
| | (.40) | (.32) | (.29) | (.65) | (.042) | (.034) | (.072) | (.040) | (.044) |
| Life expectancy | **.030**$^\dagger$ | ***−.046*** | −.018 | −.015 | **.22**$^\dagger$ | ***−.34*** | −.060 | .030 | ***−.031*** |
| | (.008) | (.019) | (.024) | (.015) | (.060) | (.14) | (.059) | (.025) | (.013) |
| Freedom of choice | **1.03**$^\star$ | **.83**$^\dagger$ | **.65**$^\dagger$ | **1.70**$^\star$ | **.13**$^\star$ | **.11**$^\dagger$ | **.33**$^\star$ | **.12**$^\star$ | **.15**$^\dagger$ |
| | (.33) | (.24) | (.20) | (.54) | (.042) | (.031) | (.10) | (.036) | (.045) |
| Giving | **.92**$^\dagger$ | .26 | *.31*$^+$ | .016 | **.13**$^\dagger$ | .036 | .003 | *.058*$^+$ | .048 |
| | (.27) | (.20) | (.17) | (.39) | (.038) | (.028) | (.080) | (.031) | (.037) |
| Corruption | ***−.67*** | *−.43*$^+$ | −.18 | ***−1.01*** | ***−.11*** | *−.070*$^+$ | ***−.17*** | −.028 | *−.065*$^+$ |
| | (.29) | (.25) | (.28) | (.48) | (.047) | (.040) | (.081) | (.041) | (.037) |
| model | XS | FE | FD | 2P | XS | FE | 2P | FD | FE |
| normalization | | | | | XS | XS | 2P | FD | FD |
| Year f.e. | 12 | 12 | 12 | | 12 | 12 | | 12 | 12 |
| $N_{clusters}$ | 149 | 149 | 139 | | 149 | 149 | | 139 | 149 |
| obs. | 1212 | 1212 | 971 | 108 | 1212 | 1212 | 108 | 971 | 1212 |
| $R^2$(adj) | .755 | .130 | .060 | .347 | .755 | .130 | .347 | .064 | .130 |
| $R^2$(overall) | | .64 | | | | .64 | | | .64 |

Significance: **0.1%**$^\dagger$ **1%**$^\star$ ***5%*** *10%*$^+$

Supplementary Table 4: **Estimates of life evaluations**. Estimates of models accounting for cross-sectional (XS) and temporal (FE, FD, 2P) variation in life evaluations are shown (columns 1–4), along with standardized coefficient versions (columns 5-9) of the same estimates. The estimates in column (4) are used for our main projections to relate changes in our predictor variables to changes in life evaluations. The relative importance of those predictor variables for explaining variation in life evaluations can be gauged by looking at the standardized coefficients. We prefer column (7) as a measure of explanatory power for changes over time.

| | Affect balance | | | | | | | | |
|---|---|---|---|---|---|---|---|---|---|
| | (1) | (2) | (3) | (4) | (5) | (6) | (7) | (8) | (9) |
| log(GDP/capita) | −.018 | .031 | ***.13*** | ***.13*** | −.14 | .24 | ***.23*** | **.100**$^\dagger$ | .016 |
| | (.013) | (.043) | (.050) | (.059) | (.10) | (.34) | (.11) | (.028) | (.023) |
| Social support | **.50**$^\dagger$ | **.27**$^\dagger$ | **.20**$^\dagger$ | .049 | **.40**$^\dagger$ | **.22**$^\dagger$ | .033 | **.15**$^\dagger$ | **.19**$^\dagger$ |
| | (.071) | (.070) | (.057) | (.15) | (.056) | (.055) | (.098) | (.042) | (.049) |
| Life expectancy | −.0004 | ***−.008*** | −.005 | −.008 | −.020 | ***−.43*** | −.19 | .038 | ***−.027*** |
| | (.002) | (.004) | (.005) | (.006) | (.11) | (.21) | (.13) | (.026) | (.013) |
| Freedom of choice | **.40**$^\dagger$ | **.16**$^\star$ | **.10**$^\star$ | .035 | **.38**$^\dagger$ | **.15**$^\star$ | .040 | **.11**$^\star$ | **.15**$^\star$ |
| | (.067) | (.049) | (.036) | (.10) | (.064) | (.047) | (.12) | (.036) | (.046) |
| Giving | **.16**$^\dagger$ | .052 | .027 | **.18**$^\star$ | **.17**$^\dagger$ | .054 | **.23**$^\star$ | .028 | .050 |
| | (.044) | (.049) | (.040) | (.071) | (.046) | (.051) | (.087) | (.042) | (.047) |
| Corruption | *−.074*$^+$ | −.048 | −.026 | −.15 | *−.090*$^+$ | −.059 | −.15 | −.024 | −.037 |
| | (.038) | (.045) | (.048) | (.093) | (.047) | (.055) | (.094) | (.039) | (.035) |
| model | XS | FE | FD | 2P | XS | FE | 2P | FD | FE |
| normalization | | | | | XS | XS | 2P | FD | FD |
| Year f.e. | 12 | 12 | 12 | | 12 | 12 | | 12 | 12 |
| $N_{clusters}$ | 149 | 149 | 139 | | 149 | 149 | | 139 | 149 |
| obs. | 1208 | 1208 | 965 | 108 | 1208 | 1208 | 108 | 965 | 1208 |
| $R^2$(adj) | .468 | .083 | .043 | .151 | .468 | .083 | .151 | .049 | .083 |
| $R^2$(overall) | | .28 | | | | .28 | | | .28 |

Significance: **0.1%**$^\dagger$ **1%**$^\star$ ***5%*** *10%*$^+$

Supplementary Table 5: **Estimates of positive affect**. See Supplementary Table 4.

| | (1) | (2) | (3) | (4) | + affect<br>(5) | (6) | (7) | (8) | (9) |
|---|---|---|---|---|---|---|---|---|---|
| log(GDP/capita) | −.003 | **.070**$^{\star}$ | *.076*$^{+}$ | **.10**$^{\star}$ | −.037 | **.77**$^{\star}$ | **.31**$^{\star}$ | ***.074*** | **.056**$^{\star}$ |
| | (.010) | (.022) | (.040) | (.033) | (.11) | (.24) | (.100) | (.037) | (.017) |
| Social support | **.24**$^{\dagger}$ | ***.089*** | .033 | −.024 | **.27**$^{\dagger}$ | ***.099*** | −.028 | .035 | ***.094*** |
| | (.052) | (.041) | (.036) | (.095) | (.058) | (.046) | (.11) | (.040) | (.044) |
| Life expectancy | .0006 | −.002 | −.002 | *−.004*$^{+}$ | .046 | −.14 | *−.16*$^{+}$ | .009 | −.009 |
| | (.001) | (.001) | (.003) | (.002) | (.11) | (.11) | (.093) | (.018) | (.008) |
| Freedom of choice | **.33**$^{\dagger}$ | **.11**$^{\dagger}$ | **.090**$^{\dagger}$ | *.092*$^{+}$ | **.44**$^{\dagger}$ | **.15**$^{\dagger}$ | *.18*$^{+}$ | **.13**$^{\dagger}$ | **.16**$^{\dagger}$ |
| | (.042) | (.029) | (.027) | (.050) | (.056) | (.039) | (.099) | (.040) | (.041) |
| Giving | **.16**$^{\dagger}$ | **.085**$^{\star}$ | **.077**$^{\star}$ | ***.11*** | **.24**$^{\dagger}$ | **.13**$^{\star}$ | ***.22*** | **.12**$^{\star}$ | **.12**$^{\star}$ |
| | (.031) | (.032) | (.028) | (.043) | (.047) | (.048) | (.090) | (.042) | (.046) |
| Corruption | .027 | .002 | −.009 | .008 | .046 | .003 | .013 | −.012 | .002 |
| | (.029) | (.030) | (.031) | (.057) | (.049) | (.052) | (.097) | (.037) | (.035) |
| model | XS | FE | FD | 2P | XS | FE | 2P | FD | FE |
| normalization | | | | | XS | XS | 2P | FD | FD |
| Year f.e. | 12 | 12 | 12 | | 12 | 12 | | 12 | 12 |
| $N_{clusters}$ | 149 | 149 | 139 | | 149 | 149 | | 139 | 149 |
| obs. | 1209 | 1209 | 966 | 108 | 1209 | 1209 | 108 | 966 | 1209 |
| $R^2$(adj) | .482 | .089 | .036 | .219 | .482 | .089 | .219 | .037 | .089 |
| $R^2$(overall) | | .23 | | | | .23 | | | .23 |

Significance: **0.1%**$^{\dagger}$ **1%**$^{\star}$ ***5%*** *10%*$^{+}$

Supplementary Table 6: **Estimates of negative affect**. See Supplementary Table 4.

| | | | | | − affect | | | | |
|---|---|---|---|---|---|---|---|---|---|
| | (1) | (2) | (3) | (4) | (5) | (6) | (7) | (8) | (9) |
| log(GDP/capita) | *.014*$^{+}$ | .040 | *−.047*$^{+}$ | −.026 | *.22*$^{+}$ | .61 | −.070 | **−.075**$^{\dagger}$ | .033 |
| | (.008) | (.031) | (.025) | (.040) | (.12) | (.48) | (.11) | (.023) | (.026) |
| Social support | **−.26**$^{\dagger}$ | **−.18**$^{\dagger}$ | **−.17**$^{\dagger}$ | −.064 | **−.40**$^{\dagger}$ | **−.29**$^{\dagger}$ | −.066 | **−.20**$^{\dagger}$ | **−.20**$^{\dagger}$ |
| | (.046) | (.052) | (.047) | (.092) | (.072) | (.080) | (.095) | (.055) | (.057) |
| Life expectancy | .0009 | *.006*$^{+}$ | .003 | .004 | .10 | *.65*$^{+}$ | .13 | *−.050*$^{+}$ | *.032*$^{+}$ |
| | (.001) | (.003) | (.003) | (.004) | (.11) | (.34) | (.14) | (.029) | (.017) |
| Freedom of choice | −.072 | −.043 | −.015 | .058 | −.14 | −.080 | .10 | −.022 | −.062 |
| | (.046) | (.035) | (.022) | (.073) | (.085) | (.065) | (.13) | (.035) | (.051) |
| Giving | .004 | .034 | *.048*$^{+}$ | −.078 | .008 | .070 | −.15 | *.080*$^{+}$ | .050 |
| | (.031) | (.029) | (.025) | (.049) | (.063) | (.061) | (.094) | (.041) | (.044) |
| Corruption | **.10**$^{\dagger}$ | *.049*$^{+}$ | .017 | **.15**$^{\star}$ | **.24**$^{\dagger}$ | *.12*$^{+}$ | **.23**$^{\star}$ | .026 | *.060*$^{+}$ |
| | (.025) | (.027) | (.030) | (.056) | (.061) | (.065) | (.087) | (.038) | (.033) |
| model | XS | FE | FD | 2P | XS | FE | 2P | FD | FE |
| normalization | | | | | XS | XS | 2P | FD | FD |
| Year f.e. | 12 | 12 | 12 | | 12 | 12 | | 12 | 12 |
| $N_{\text{clusters}}$ | 149 | 149 | 139 | | 149 | 149 | | 139 | 149 |
| obs. | 1211 | 1211 | 970 | 108 | 1211 | 1211 | 108 | 970 | 1211 |
| $R^2$(adj) | .221 | .088 | .046 | .045 | .221 | .088 | .045 | .052 | .088 |
| $R^2$(overall) | | .002 | | | | .002 | | | .002 |

Significance: **0.1%**$^{\dagger}$ **1%**$^{\star}$ ***5%*** *10%*$^{+}$

Supplementary Table 7: **Estimates of affect balance**. See Supplementary Table 4.

| | Life today | | | | | | | |
|---|---|---|---|---|---|---|---|---|
| | (1) | (2) | (3) | (4) | (5) | (6) | (7) | (8) |
| log(GDP/capita) | *.19*$^{+}$ | *.21*$^{+}$ | *.20*$^{+}$ | ***.24*** | *.19*$^{+}$ | *.19*$^{+}$ | *.21*$^{+}$ | ***.24*** |
| | (.11) | (.11) | (.11) | (.11) | (.11) | (.11) | (.11) | (.11) |
| Social support | **.29**$^{\dagger}$ | ***.21*** | **.29**$^{\dagger}$ | **.27**$^{\dagger}$ | **.29**$^{\dagger}$ | **.30**$^{\dagger}$ | **.29**$^{\dagger}$ | *.18*$^{+}$ |
| | (.072) | (.098) | (.072) | (.073) | (.072) | (.079) | (.072) | (.10) |
| Life expectancy | −.060 | −.055 | −.062 | *−.10*$^{+}$ | −.061 | −.064 | −.064 | −.091 |
| | (.059) | (.058) | (.061) | (.063) | (.059) | (.059) | (.060) | (.062) |
| Freedom of choice | **.33**$^{\star}$ | **.33**$^{\star}$ | **.33**$^{\star}$ | **.48**$^{\dagger}$ | **.33**$^{\star}$ | **.32**$^{\star}$ | **.30**$^{\star}$ | **.57**$^{\dagger}$ |
| | (.10) | (.11) | (.10) | (.12) | (.10) | (.11) | (.11) | (.13) |
| Giving | .003 | −.005 | .005 | .020 | .006 | .004 | .030 | −.0003 |
| | (.080) | (.082) | (.083) | (.076) | (.081) | (.081) | (.10) | (.072) |
| Corruption | ***−.17*** | ***−.18*** | *−.18*$^{+}$ | ***−.17*** | *−.15*$^{+}$ | ***−.17*** | ***−.17*** | −.032 |
| | (.081) | (.081) | (.11) | (.083) | (.087) | (.082) | (.082) | (.096) |
| log(GDP/capita) × Social support | | .11 | | | | | | .14 |
| | | (.10) | | | | | | (.097) |
| log(GDP/capita) × Corruption | | | .015 | | | | | *−.22*$^{+}$ |
| | | | (.11) | | | | | (.12) |
| log(GDP/capita) × Freedom of choice | | | | **−.26**$^{\star}$ | | | | **−.39**$^{\dagger}$ |
| | | | | (.099) | | | | (.12) |
| Social support × Corruption | | | | | .037 | | | |
| | | | | | (.064) | | | |
| Social support × Freedom of choice | | | | | | −.045 | | |
| | | | | | | (.072) | | |
| Freedom of choice × Giving | | | | | | | −.077 | |
| | | | | | | | (.097) | |
| model | 2P | 2P | 2P | 2P | 2P | 2P | 2P | 2P |
| AIC | 267 | 268 | 269 | 263 | 269 | 269 | 268 | 263 |
| obs. | 108 | 108 | 108 | 108 | 108 | 108 | 108 | 108 |
| $R^2$(adj) | .347 | .346 | .340 | .376 | .341 | .342 | .345 | .387 |

Significance: **0.1%**$^{\dagger}$ **1%**$^{\star}$ ***5%*** *10%*$^{+}$

Supplementary Table 8: **Estimates of life evaluations including interaction terms**. Column (1) reproduces our preferred 2P model (column 7 of Supplementary Table 4). Columns (2) through (7) are the same but with the addition of one interaction term each. Column (8) includes all terms between material and non-material predictor variables. All coefficients are presented normalized to 2P variation.

| | Affect balance | + affect | − affect | log(GDP/capita) | Life expectancy | Corruption | Freedom of choice | Giving | Social support |
|---|---|---|---|---|---|---|---|---|---|
| Life today | **.51**$^{\dagger}$ ($<10^{-5}$) | **.59**$^{\dagger}$ ($<10^{-5}$) | **−.21**$^{\star}$ (.007) | **.83**$^{\dagger}$ ($<10^{-5}$) | **.77**$^{\dagger}$ ($<10^{-5}$) | **−.47**$^{\dagger}$ ($<10^{-5}$) | **.57**$^{\dagger}$ ($<10^{-5}$) | **.23**$^{\star}$ (.004) | **.75**$^{\dagger}$ ($<10^{-5}$) |
| Affect balance | | **.90**$^{\dagger}$ ($<10^{-5}$) | **−.78**$^{\dagger}$ ($<10^{-5}$) | **.28**$^{\dagger}$ (.0004) | **.23**$^{\star}$ (.004) | **−.37**$^{\dagger}$ ($<10^{-5}$) | **.64**$^{\dagger}$ ($<10^{-5}$) | **.35**$^{\dagger}$ (1e-05) | **.49**$^{\dagger}$ ($<10^{-5}$) |
| $+$ affect | | | **−.44**$^{\dagger}$ ($<10^{-5}$) | **.35**$^{\dagger}$ (1e-05) | **.30**$^{\dagger}$ (.0002) | **−.33**$^{\dagger}$ (3e-05) | **.69**$^{\dagger}$ ($<10^{-5}$) | **.42**$^{\dagger}$ ($<10^{-5}$) | **.49**$^{\dagger}$ ($<10^{-5}$) |
| $-$ affect | | | | −.075 (.36) | −.057 (.48) | **.28**$^{\dagger}$ (.0004) | **−.33**$^{\dagger}$ (2e-05) | −.12 (.15) | **−.31**$^{\dagger}$ (7e-05) |
| log(GDP/capita) | | | | | **.83**$^{\dagger}$ ($<10^{-5}$) | **−.39**$^{\dagger}$ ($<10^{-5}$) | **.41**$^{\dagger}$ ($<10^{-5}$) | −.014 (.87) | **.71**$^{\dagger}$ ($<10^{-5}$) |
| Life expectancy | | | | | | **−.29**$^{\dagger}$ (.0003) | **.36**$^{\dagger}$ (1e-05) | .059 (.47) | **.63**$^{\dagger}$ ($<10^{-5}$) |
| Corruption | | | | | | | **−.54**$^{\dagger}$ ($<10^{-5}$) | **−.32**$^{\dagger}$ (8e-05) | **−.22**$^{\star}$ (.006) |
| Freedom of choice | | | | | | | | **.41**$^{\dagger}$ ($<10^{-5}$) | **.42**$^{\dagger}$ ($<10^{-5}$) |
| Giving | | | | | | | | | .087 (.29) |

Significance: **0.1%**$^{\dagger}$ **1%**$^{\star}$ ***5%*** *10%*$^{+}$

Supplementary Table 9: **Pairwise correlations of levels**. Pearson correlation coefficients for our country-mean values of our main variables

| | Affect balance | + affect | − affect | log(GDP/capita) | Life expectancy | Corruption | Freedom of choice | Giving | Social support |
|---|---|---|---|---|---|---|---|---|---|
| Life today | **.41**† | **.33**† | **−.32**† | **.39**† | −.043 | **−.30**⋆ | **.46**† | .11 | **.43**† |
| | ($<10^{-5}$) | (.0003) | (.0003) | (1e-05) | (.64) | (.001) | ($<10^{-5}$) | (.22) | ($<10^{-5}$) |
| Affect balance | | **.78**† | **−.80**† | **.30**⋆ | −.11 | ***−.21*** | *.17*+ | *.17*+ | *.15*+ |
| | | ($<10^{-5}$) | ($<10^{-5}$) | (.001) | (.24) | (.026) | (.060) | (.072) | (.091) |
| + affect | | | **−.25**⋆ | **.39**† | −.023 | −.10 | **.29**⋆ | ***.22*** | .064 |
| | | | (.005) | (1e-05) | (.80) | (.29) | (.002) | (.018) | (.48) |
| − affect | | | | −.091 | .14 | ***.23*** | −.002 | −.056 | *−.18*+ |
| | | | | (.32) | (.12) | (.016) | (.98) | (.55) | (.052) |
| log(GDP/capita) | | | | | .14 | −.15 | **.39**† | .058 | **.27**⋆ |
| | | | | | (.13) | (.13) | (2e-05) | (.54) | (.003) |
| Life expectancy | | | | | | .011 | .093 | .082 | −.025 |
| | | | | | | (.90) | (.32) | (.38) | (.79) |
| Corruption | | | | | | | **−.39**† | −.061 | −.008 |
| | | | | | | | (2e-05) | (.53) | (.94) |
| Freedom of choice | | | | | | | | .14 | .061 |
| | | | | | | | | (.14) | (.51) |
| Giving | | | | | | | | | .063 |
| | | | | | | | | | (.50) |

Significance: **0.1%**† **1%**⋆ ***5%*** *10%*+

Supplementary Table 10: **Pairwise correlations of changes**. Pearson correlation coefficients for two-period changes of our main variables

| | Life today | | |
|---|---|---|---|
| | (1) | (2) | (3) |
| log(GDP/capita) | **.35**† | **.23**† | ***.19*** |
| | (.062) | (.069) | (.083) |
| Social support | **2.2**† | **1.59**† | **3.1**† |
| | (.40) | (.35) | (.53) |
| Life expectancy | **.030**† | −.012 | .009 |
| | (.008) | (.011) | (.035) |
| Freedom of choice | **1.03**⋆ | ***.77*** | 1.60 |
| | (.33) | (.32) | (1.04) |
| Corruption | ***−.67*** | .25 | .18 |
| | (.29) | (.33) | (.53) |
| Giving | **.92**† | 1.09 | .12 |
| | (.27) | (.73) | (.50) |
| model | XS | XS | XS |
| normalization | | | |
| region | All countries | Sub-Saharan Africa | C.I.S. |
| Year f.e. | 12 | 12 | 12 |
| obs. | 1212 | 284 | 116 |
| $R^2$(adj) | .755 | .335 | .629 |
| $N_{clusters}$ | 149 | 39 | 11 |

Significance: **0.1%**† **1%**⋆ ***5%*** *10%*+

Supplementary Table 11: **XS model estimated for regions.** Column 1 is repeated from Supplementary Table 4. Column 2 is the same model estimated for the subset of countries in sub-Saharan Africa, and Column 3 is the same but estimated for the Commonwealth of Independent States, ie former nations of the Soviet Union.

| scenario | min | max | mean (nat'l) | mean (pop'n) | 2016value (nat'l) | 2016value (pop'n) |
|---|---|---|---|---|---|---|
| Material trends (OECD optimistic) | 4.24 | 7.69 | 6.05 | 6.04 | 5.35 | 5.24 |
| Material trends (OECD pessimistic) | 3.52 | 7.73 | 5.72 | 5.66 | 5.35 | 5.24 |
| Material trends (optimistic) | 3.67 | 8.31 | 6.12 | 6.01 | 5.35 | 5.24 |
| Material trends (pessimistic) | 2.76 | 7.40 | 5.21 | 5.10 | 5.35 | 5.24 |
| Non-material trends (optimistic) | 4.50 | 8.61 | 6.86 | 6.84 | 5.35 | 5.24 |
| Non-material trends (pessimistic) | 1.09 | 5.57 | 3.52 | 3.44 | 5.35 | 5.24 |

Supplementary Table 12: Scenario projections using two-period (2P) coefficients

| scenario | min | max | mean (nat'l) | mean (pop'n) | 2016value (nat'l) | 2016value (pop'n) |
|---|---|---|---|---|---|---|
| Material trends (OECD optimistic) | 3.97 | 7.40 | 5.91 | 5.95 | 5.35 | 5.24 |
| Material trends (OECD pessimistic) | 3.47 | 7.72 | 5.77 | 5.74 | 5.35 | 5.24 |
| Material trends (optimistic) | 3.56 | 8.21 | 6.01 | 5.90 | 5.35 | 5.24 |
| Material trends (pessimistic) | 2.60 | 7.24 | 5.05 | 4.94 | 5.35 | 5.24 |
| Non-material trends (optimistic) | 4.15 | 8.03 | 6.22 | 6.16 | 5.35 | 5.24 |
| Non-material trends (pessimistic) | 1.83 | 6.39 | 4.27 | 4.18 | 5.35 | 5.24 |

Supplementary Table 13: Scenario projections using fixed-effect (FE) coefficients

| scenario | min | max | mean (nat'l) | mean (pop'n) | 2016value (nat'l) | 2016value (pop'n) |
|---|---|---|---|---|---|---|
| Material trends (OECD optimistic) | 4.36 | 8.27 | 6.39 | 6.34 | 5.35 | 5.24 |
| Material trends (OECD pessimistic) | 3.39 | 7.78 | 5.71 | 5.62 | 5.35 | 5.24 |
| Material trends (optimistic) | 3.98 | 8.63 | 6.43 | 6.32 | 5.35 | 5.24 |
| Material trends (pessimistic) | 2.98 | 7.63 | 5.43 | 5.32 | 5.35 | 5.24 |
| Non-material trends (optimistic) | 4.83 | 8.72 | 6.97 | 6.93 | 5.35 | 5.24 |
| Non-material trends (pessimistic) | 0.99 | 5.51 | 3.42 | 3.33 | 5.35 | 5.24 |

Supplementary Table 14: Scenario projections using cross-section (XS) coefficients

# Supplementary Note 1 (Descriptive statistics)

Our main text focuses on one primary measure of subjective well-being (SWB), namely the cognitive evaluation of life question called "Cantril's Ladder." However, this supplement includes analysis of two other dimensions of SWB, positive and negative affect, and the difference between them, "affect balance." For more details, again see [8].

Supplementary Table 3 shows summary statistics for these data, while Supplementary Table 2 shows summary statistics for country changes between successive years (first differences, or FD), where they are available. Supplementary Table 1 shows summary statistics for differences between country means over the period 2014–2016 and corresponding means over the period 2005-2007; we call these our two-period (2P) estimates.

## Pairwise correlations

The simplest measure of the predictive relationships we explore and exploit is a bivariate correlation. Supplementary Table 9 shows pair-wise correlations (Pearson coefficients) among country means for our key variables. Supplementary Table 10 shows pair-wise correlations (Pearson coefficients) among changes in our key variables between the two periods in our two-period (2P) data.

While country averages of life evaluations are, among our predictor variables, most closely correlated (Supplementary Table 9) with levels of per capita income, *changes* in life evaluations are more closely correlated (Supplementary Table 10) with changes in social support ($R \approx .43$) or freedom ($R \approx .46$) than they are with changes in income ($R \approx .39$).

# Supplementary Note 2 (Model estimates)

The four models, described in Methods, are identified as "XS" (static model), "FE" (annual changes, fixed effects), "FD" (annual changes, first differences), and "2P" (two-period) and estimated for cognitive evaluations of life in Supplementary Table 4. In our favored dynamic model (2P), changes in "giving" and life expectancy do not predict significant changes in average life evaluation. Tables 5–7 report analogous estimates for the other measures of SWB. In each case, the first four columns present estimates of raw coefficients ($b$, in the discussion above), while the remaining columns show $\beta$ coefficients standardized in one of three ways. We consider column (7) to give the best indication of the relative importance of different predictor variables for explaining changes in SWB, and we use the corresponding raw coefficients in column (4) for our primary scenario projections.

While the importance of income, as represented by standardized coefficients in our model, is lower in the dynamic model than in the cross-sectional one, the underlying effect size is actually larger. That is, a 10% increase in income per capita accounts for an increase in life evaluations of 0.066 (on an 11-point scale) with the dynamic 2P model (i.e., within a country), while a 10% difference in income per capita accounts for a difference of only 0.035 in the static model (i.e., across countries). In the case of our models of year-to-year changes (FD and FE), the dynamic effect is larger still. The latter finding may reflect the impacts of economic fluctuations in the business cycle and the 2008 financial crisis, which our two-period model expressly avoids. More generally, the quantitative difference we observe is consistent with other evidence that income tends to affect well-being more strongly in the short term than over the long run [e.g., 19, 23]. Despite this possible exaggeration of the effect of GDP, which may overestimate the SWB gains to be made from a longer-term rise of GDP, we use the time-series model coefficients in the scenarios that follow.

## Estimates for country regions

In light of the hypothesis that life evaluations should depend more on income in places with lower incomes, Supplementary Table 11 shows estimates of the XS model for sub-Saharan Africa and for the Commonwealth of Independent States. Both regions have a sufficient number of countries to carry out the estimate; however, fewer coefficients are found to be statistically significant with the smaller samples. Contrary to the hypothesis, the ratio of coefficients on GDP/capita and social support are similar or smaller in the sub-regions as in the global estimate. For this reason, and to achieve reasonable precision in our estimates, we do not split up the planet according

to levels of development in the primary estimates of our models.

## Supplementary Note 3 (Robustness tests of interaction effects)

In order to test for interaction (sometimes called moderation) effects between our predictor variables, we focus on the four statistically significant variables in our dynamic 2P model used for our primary analysis. Three of these variables are "non-material", while one, log(GDP/capita), is "material". Therefore, of the pair-wise combinations among them, three interactions could indicate a problem with our separation of non-material scenarios from material scenarios, while the remaining three interactions concern only the specification within the non-material model.

To best assess the size of any interaction effects in our baseline model, we introduce six new normalized variables (i.e., all pair-wise combinations of four predictor variables) one-by-one into the normalized-coefficient 2P model. As shown in columns (2) to (7) of Supplementary Table 8, we find non-significant effects for all but one of them. In the case of log(GDP/capita)$\times$Freedom of choice (column 4) a significant interaction effect is estimated. Apart from the interaction itself, there is no qualitative (nor significant quantitative) change in the other estimated parameters, as compared with our baseline model (shown in column 1). Because we recognize that our predictor variables are only proxies for material and non-material drivers of life quality, and that they are likely to exhibit multicollinearity, we interpret these findings as relatively reassuring in that our projections, though necessarily crude, represent a reasonable approach. In our 2P analysis, when only the material variables or the non-material variables, but not both, are changing in any scenario, the significant interaction term will remain zero by construction.

We also estimate a version of the model including all the interactions between our (statistically significant) material and non-material RHSvs. This estimate is shown in column (8) of Supplementary Table 8. In this case, there is a particularly significant interaction between GDP/capita and Freedom of choice.

We emphasize that our data set (119 countries) is too small to conduct model selection procedures, and that comparing models for quality of fit is not a well-constrained undertaking. A formal test of interaction effects should likely stop at a test of significance of interaction coefficients in columns (2) to (7) in order to limit the likelihood of finding spurious relationships. Were the data extensive enough to expand the model beyond the original 6 predictors, numerous other specifications accommodating nonlinear terms and other available national-level variables would be

equally reasonable candidates to specifications that are expanded using interaction terms. For this reason, we constrained our base model to a specification already established in the literature.

Nevertheless, in order to compare the alternate specifications, Supplementary Table 8 shows the Akaike information criterion (AIC) for each model. Lower values of the AIC indicate a better tradeoff between goodness-of-fit and model simplicity. Only two models, in columns (4) and (8), have a lower AIC than our baseline model (column 1). To assess the impact on our projections of including interaction terms, we repeated our 2P projection calculations using these two models. The results are shown in Supplementary Figure 1. The top panel shows our preferred projection from the main text. The second panel shows projections based on column (4) of Supplementary Table 8, which includes the interaction term between GDP/capita and Freedom of choice. The primary difference from our preferred model is that the projected range of non-material projection outcomes is even larger than in our preferred version. In contrast, the lower panel shows the projections based on column (8), in which all the "conflating" interaction terms between material and non-material statistically significant predictor variables are included. In this case, there is a reduction in the 90/10 range of projections in the non-material scenario, particularly in the outcome of the most optimistic non-material trends. However, the 30/70 range remains quite similar to our preferred model, and our conclusions remain robust in that the range of our projected distributions in the non-material scenario is far greater than those of the two material scenarios. Given that the AIC is the same as for model (4) which shows a very large range of non-material projections, we maintain our preference for the intermediate results of model (1), particularly given that it is the simplest model and therefore least prone to over-fitting.
